# Comparative Seroprevalence of *Brucella* Antibodies in Apparently Healthy Sheep and Goats in a Rural District of Central Nigeria

**DOI:** 10.1155/vmi/5095222

**Published:** 2026-10-19

**Authors:** Martin Zang Pam, Alex Odoom, Charles Matthew Kakka, Fidelis Leo Latkal, Kete Clinton Waziri, Solomon Jere Dule, Suzanna Zakka Nyango, Pedung Raphael Emmanuel

**Affiliations:** ^1^ Department of Animal Health Federal College of Animal Health and Production Technology National Veterinary Research Institute (NVRI) Vom Jos Plateau Nigeria nvri.gov.ng; ^2^ Department of Epidemiology and Disease Control School of Public Health University of Ghana Legon Accra Ghana ug.edu.gh; ^3^ Department of Medical Microbiology University of Ghana Medical School Korle Bu Accra Ghana ug.edu.gh; ^4^ Department of General Studies Federal College of Animal Health and Production Technology National Veterinary Research Institute (NVRI) Vom Plateau Nigeria nvri.gov.ng

**Keywords:** *Brucella* antibodies, brucellosis, Central Nigeria, seroprevalence, small ruminants

## Abstract

**Background:**

Brucellosis, caused by bacterial pathogens of the genus *Brucella*, is a chronic zoonotic disease affecting small ruminants such as sheep and goats. This study evaluated and compared the seroprevalence of *Brucella* antibodies among apparently healthy sheep and goats in Vwang District, Jos South Local Government Area, Plateau State, Nigeria.

**Methodology:**

A cross‐sectional study design was employed from May to June 2024. A total of 140 serum samples collected from goats (*n* = 77) and sheep (*n* = 63) using systematic random sampling across four administrative wards were screened for *Brucella* antibodies using the Rose Bengal Plate Test (RBPT) and confirmed via the Complement Fixation Test (CFT).

**Results:**

Overall, 8 out of 140 samples (5.71%, 95% confidence interval [CI]: 2.50%–10.95%) were reactive on RBPT screening. Upon confirmatory testing with CFT, 6 samples were validated, yielding an overall CFT‐confirmed seroprevalence of 4.29% (95% CI: 1.59−9.09%). Seroprevalence was higher in sheep (6.35%, 95% CI: 1.76−15.47%) than in goats (2.60%, 95% CI: 0.32−9.07%); higher in females (4.95%, 95% CI: 1.63−11.18%) than in males (2.56%, 95% CI: 0.06−13.48%); and restricted to animals older than 18 months (6.74%, 95% CI: 2.51−14.10%) compared with younger ones (0.00%, 95% CI: 0.00−6.98%). Geographically, the Turu B Ward recorded the highest seroprevalence (5.88%, 95% CI: 0.72−19.68%). None of the demographic associations reached statistical significance (*p* > 0.05).

**Conclusion:**

This study provides serological evidence of *Brucella* exposure among apparently healthy sheep and goats in Vwang District, Central Nigeria. Although seroprevalence differences across species, sex, age, and location were not statistically significant, the detection of antibodies in nonvaccinated, clinically healthy livestock highlights the value of routine serological monitoring in rural agrarian communities.

## 1. Introduction

Brucellosis is a chronic zoonotic disease of global livestock and public health importance caused by bacterial pathogens of the genus *Brucella* [1, 2]. These bacteria infect both phagocytic and nonphagocytic host cells, facilitating persistence and systemic dissemination across multiple organ systems [3, 4]. Transmission among livestock occurs primarily through direct contact with or ingestion of contaminated reproductive discharges, aborted fetuses, milk, urine, or placental membranes [5, 6]. Infection can also be transmitted from clinically healthy infected animals shedding the organism in secretions [1].

At least nine *Brucella* species affect terrestrial and marine animals, with *Brucella melitensis* (primarily infecting goats and sheep) and *Brucella ovis* (causing contagious epididymitis in rams) being the primary species of concern in small ruminants [7–9]. These pathogens cause reproductive failure, abortion, infertility, and reduced milk yield, resulting in severe economic losses in livestock‐dependent rural communities [6]. Human brucellosis is caused primarily by zoonotic species, notably *B. melitensis*, *B. abortus*, and *B. suis*, which infect livestock owners, veterinarians, and abattoir workers through occupational contact or consumption of unpasteurized dairy products [1].

Serological screening methods such as the Rose Bengal Plate Test (RBPT) utilize smooth *Brucella abortus* biotype 1 antigen containing smooth lipopolysaccharide (S‐LPS) O‐polysaccharide chain epitopes [10]. Because *B. abortus* shares major O‐antigenic epitopes with *B. melitensis*, RBPT serves as an effective screening assay for smooth *Brucella* species, although cross‐reactivity with other Gram‐negative bacteria possessing shared O‐polysaccharide structures (e.g., *Yersinia enterocolitica* O:9) can occur [11, 12]. Consequently, serial testing using a highly specific secondary assay such as the Complement Fixation Test (CFT) is required for diagnostic confirmation according to World Organisation for Animal Health (WOAH) guidelines [13].

In Nigeria, brucellosis has been documented since the 1970s, with reported seroprevalence rates ranging widely from 0.20% to 79.70% depending on geographical region, production system, and diagnostic criteria [14]. In rural agrarian settings such as Vwang District (Vom) in Plateau State, small ruminant farming represents a vital household livelihood asset. While bovine brucellosis has been investigated in Plateau State [15], comparative epidemiological data regarding *Brucella* species exposure in apparently healthy sheep and goats remain limited. Therefore, this cross‐sectional study aimed to provide baseline data on and compare the seroprevalence of *Brucella* antibodies in apparently healthy sheep and goats in Vwang District, Central Nigeria, using a serial RBPT screening and CFT confirmation testing protocol.

## 2. Material and Methods

### 2.1. Study Area

The study was conducted in Vwang District (Vom), situated between latitude 9.72°–9.74 N and longitude 8.77°–8.80 E in Jos South Local Government Area (LGA), Plateau State, Central Nigeria (Figure 1). Positioned on the Jos Plateau at an elevation exceeding 1200 m above sea level, Vwang experiences a tropical highland climate with mean annual temperatures ranging between 15°C and 22°C. Small ruminants in Vwang District are predominantly owned by rural households and reared under extensive or semi‐intensive scavenging production systems.

**FIGURE 1:**
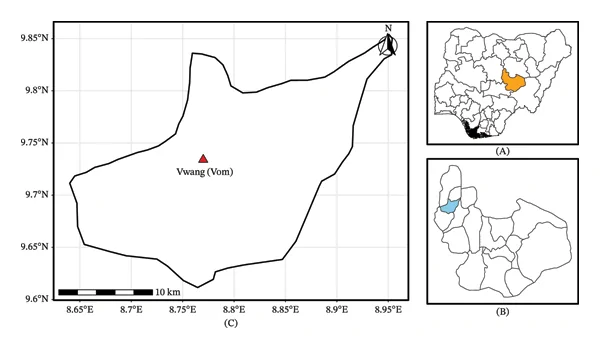
Geographic location of Vwang (Vom) District within Jos South Local Government Area (LGA), Plateau State, north‐central Nigeria. Panel A depicts the location of Plateau State within Nigeria; Panel B shows Jos South LGA within Plateau State; Panel C highlights Vwang District (9.72°–9.74 N, 8.77°–8.80 E), where livestock sampling was conducted (administrative boundary shapefiles obtained from NASRDA/DIVA‐GIS mapping databases).

### 2.2. Study Design

The work utilized a cross‐sectional research approach carried out from May to June 2024.

### 2.3. Study Population and Eligibility Criteria

The study population comprised indigenous breeds of small ruminants, specifically West African Dwarf goats, Red Sokoto goats, and West African Dwarf sheep, reared in smallholder herds across Vwang District. Eligible animals included clinically healthy male and female goats and sheep aged > 6 months that exhibited no overt clinical signs of systemic illness or active reproductive disorders upon physical examination. Animal age was determined by dentition examination (eruption of permanent incisors) combined with owner information and management history, categorizing animals into young (6–18 months) and adult (> 18 months).

Assessment of reproductive health was conducted during presampling screening: Livestock owners and herdsmen were questioned regarding flock and individual history, and any female animal with a documented or reported history of abortion, stillbirth, retained placenta, or active vaginal discharge within the preceding 6 months was excluded from the study. Because the study objective was specifically to assess baseline serological exposure in clinically healthy, non‐aborting animals, detailed lifetime reproductive records (such as parity or cumulative lifetime breeding events) were not documented for multivariable modeling. Similarly, *Brucella* livestock vaccination is not practiced in smallholder systems in this district; vaccination status was verified during presampling herd interviews, and any flock with prior vaccine exposure was excluded.

### 2.4. Sample Size Determination and Sampling Technique

#### 2.4.1. Sample Size Calculation

The target sample size was calculated using Cochran’s formula for cross‐sectional prevalence studies in animal populations:

(1) n=Z2 · p1−pd2,

where *Z* = 1.96 corresponds to the standard normal distribution at a 95% confidence level (1 − *α* = 0.95).*p* = 0.10 represents the anticipated baseline *Brucella* antibody seroprevalence in clinically healthy, nonvaccinated small ruminants in an unstudied rural district (reflecting lower‐bound estimates from regional non‐outbreak field surveys). *d* = 0.05 represents the acceptable absolute margin of error (5% precision) at the 95% confidence level.

Substituting these parameters into the formula yielded a minimum required sample size of

(2) n=138.3140≈ animals.

#### 2.4.2. Post Hoc Precision Verification

A total of 140 small ruminants (comprising 77 goats and 63 sheep) were sampled across the district. To verify the statistical adequacy of this sample size for the observed CFT‐confirmed seroprevalence (*p* = 4.29% = 0.0429), the achieved absolute precision (*d*) was calculated post hoc:

(3) d=Z×p1 −pn=0.0336 ±3.4%.

This confirms that the sample size of *N* = 140 achieved an absolute precision of ±3.4%, fulfilling standard epidemiological precision criteria (*d* ≤ 5.0%) for baseline seroprevalence estimation.

#### 2.4.3. Sampling Strategy and Animal Selection

Sampling was conducted across all four administrative wards of Vwang District: Chugwi, Turu A, Turu B, and Vwang. A two‐stage stratified sampling protocol was employed:

1. Farm/herd selection: A sampling frame of smallholder pastoral settlements and household flocks was established in each ward in collaboration with local veterinary extension agents. Within each ward, smallholder farms were selected systematically from this registry using a random starting point to ensure broad geographic representation and capture diverse management systems.
2. Individual animal selection: Within each selected herd, animals meeting inclusion criteria were corralled. A systematic random sampling procedure was implemented by assigning animals sequential numbers as they entered a holding pen or race. Animals were selected at a fixed sampling interval *k* (where *k* = *N*_herd_/*n*_target_), with the first animal chosen using a random number table between 1 and *k.* Depending on the total herd size, a proportion of 20%–50% of animals per herd was sampled (e.g., 2 animals from herds of 5, 5 from herds of 10, 7 from herds of 15, and 10 from herds of 20).

### 2.5. Sample Collection and Serum Harvesting

Blood samples (3–5 mL) were collected aseptically from selected animals via jugular venipuncture using sterile 21‐gauge needles and plain, non‐anticoagulant vacutainer tubes. Samples were allowed to clot at room temperature (22°C–25°C) for 2–4 h and held at 4°C overnight. Clotted samples were centrifuged at 1500 × g for 10 min. Clear serum was harvested using sterile Pasteur pipettes, transferred into 2.0 mL polypropylene cryovial tubes, labeled with unique animal identification numbers, and stored at −20°C prior to serological testing.

### 2.6. Laboratory Analysis

#### 2.6.1. RBPT

The RBPT is a speedy agglutination process completed within 4 minutes that is widely used and standardized for *Brucella* species screening due to its low cost and minimal equipment requirement [16]. The Rose Bengal (RB) antigen was obtained from the United Kingdom’s Veterinary Laboratories Agency, Weybridge. Laboratory tests were conducted following the procedure described by Alton et al. [17], where a 30 μL volume of serum was dispensed onto a clean ceramic tile using a micropipette, followed by the immediate addition of an equal volume of acidic‐buffered RB antigen (pH 3.65 ± 0.05). The sera and antigen complex were combined using an applicator stick and gently rocked for 4 min. The formation of any visible agglutination, granulation, or flocculation within the time span was interpreted as a positive screening reaction, whereas the complete absence of agglutination was recorded as negative. Agglutination intensity or reaction grades were not qualitatively scored, as the RBPT was deployed strictly as a qualitative, high‐sensitivity screening assay. Standard positive and negative control sera were included in each test run for quality control and assay validation.

#### 2.6.2. CFT

Serum samples that demonstrated reactivity on the RBPT screening were further subjected to the CFT for confirmation, following the protocols described by the WOAH [18]. The test was performed using standard *B. abortus* CFT antigen, guinea pig complement, and a 2% sheep red blood cell (SRBC) suspension sensitized with anti‐SRBC hemolytic serum as the indicator system. Hemolysis inhibition at a serum dilution titer of 1:4 (equivalent to 20 ICFTU/mL) or higher was considered a confirmed positive result. Standard positive and negative control sera were included in each test run to ensure diagnostic validity.

### 2.7. Data Analysis

Data were analyzed using *R* software Version 4.6.0. Overall and subgroup seroprevalence rates were reported as frequencies, percentages, and 95% confidence intervals (95% CIs) calculated using the Clopper–Pearson exact binomial method. Bivariate associations between demographic variables (species, sex, age group, and location) and CFT‐confirmed seropositivity were evaluated using univariable logistic regression to estimate crude odds ratios (CORs) with 95% CIs. For categorical comparisons with expected cell counts < 5, two‐tailed Fisher’s exact test was used to calculate exact *p*‐values. For the age comparison, where zero CFT‐positive cases were observed in the young cohort (0/51), the COR and 95% CI were estimated using the Haldane–Anscombe continuity correction by adding 0.5 to all cells of the 2 × 2 table. To control for potential mutual confounding across demographic factors, a multivariable logistic regression model was fitted, including species, sex, and age as explanatory variables, to generate adjusted odds ratios (AORs) and 95% CIs. Due to sparse event counts (*n* = 6 positive cases) and quasi‐complete separation in the age variable, the multivariable model was implemented using Firth’s penalized likelihood logistic regression with the *R* package *logistf*, which reduces small‐sample bias and yields finite parameter estimates. Significance was set at *α* = 0.05, and *p*‐values were reported to two decimal places.

### 2.8. Consent for Sample Collection

Livestock farmers were first sensitized to the importance of the study, particularly its role in generating epidemiological data to support disease surveillance efforts. Following this, livestock owners gave their informed consent before samples were collected from their animals.

### 2.9. Ethical Considerations

Ethical approval was granted by the National Veterinary Research Institute Animal Ethics Committee (NVRI/AEC Ref: AEC/02/159/24). Prior to sampling, verbal informed consent was obtained from livestock owners, and animals with a documented or reported history of *Brucella* vaccination were excluded. All procedures adhered to the International Council for Laboratory Animal Science (ICLAS) [19].

### 2.10. Patient and Public Involvement

Patients and the public were not involved in the design, conduct, reporting, or dissemination plans of this research.

## 3. Results

### 3.1. Demographics of Sampled Ruminants

A total of 140 apparently healthy small ruminants were sampled, comprising 77 goats (55.00%) and 63 sheep (45.00%). The sample included 101 females (72.14%, 95% CI: 63.94%–79.38%) and 39 males (27.86%, 95% CI: 20.62%–36.06%). By age, 89 animals (63.57%, 95% CI: 55.02%–71.53%) were older than 18 months, and 51 animals (36.43%, 95% CI: 28.47%–44.98%) were aged 6–18 months. Samples were distributed across Chugwi (*n* = 37, 26.43% [95% CI: 19.34%–34.54%]), Vwang (*n* = 35, 25.00% [95% CI: 18.07%–33.02%]), Turu A (*n* = 34, 24.29% [95% CI: 17.44%–32.25%]), and Turu B (*n* = 34, 24.29% [95% CI: 17.44%–32.25%]) wards (Table 1).

**TABLE 1:** Demographic distribution of collected small ruminant samples in Vwang District (*n* = 140).

Category	Demographic variable	Goats (caprine) *n* = 77	Sheep (ovine) *n* = 63	Frequency *n* = 140	Proportion (%) [95% CI]
Sex	Female	51 (66.2%)	50 (79.4%)	101	72.14% [63.94–79.38]
	Male	26 (33.8%)	13 (20.6%)	39	27.86% [20.62–36.06]

Age	Young (6–18 months)	25 (32.5%)	26 (41.3%)	51	36.43% [28.47–44.98]
	Old (above 18 months)	52 (67.5%)	37 (58.7%)	89	63.57% [55.02–71.53]

Location (ward)	Chugwi	20 (26.0%)	17 (27.0%)	37	26.43% [19.34–34.54]
	Turu A	19 (24.7%)	15 (23.8%)	34	24.29% [17.44–32.25]
	Turu B	18 (23.4%)	16 (25.4%)	34	24.29% [17.44–32.25]
	Vwang	20 (26.0%)	15 (23.8%)	35	25.00% [18.07–33.02]

Total	Total	77 (55.0%)	63 (45.0%)	140	100.00%

### 3.2. *Brucella* Antibody Seroprevalence in Small Ruminants

Screening via RBPT detected *Brucella* antibodies in eight of 140 animals (5.71%, 95% CI: 2.50%–10.95%). Confirmatory testing using CFT validated 6 of these reactive samples, establishing an overall CFT‐confirmed seroprevalence of 4.29% (95% CI: 1.59%–9.09%, 6/140). By species, sheep demonstrated a higher CFT seroprevalence (6.35%, 95% CI: 1.76%–15.47%, 4/63) than goats (2.60%, 95% CI: 0.32%–9.07%, 2/77). Univariable logistic regression indicated that sheep had 2.54 times higher odds of seropositivity compared with goats (OR = 2.54, 95% CI: 0.45 to 14.36; Fisher’s exact *p* = 0.41) After adjusting for sex and age in the multivariable model, the adjusted odds ratio was 2.48 (95% CI: 0.42 to 14.62; penalized likelihood *p* = 0.33), indicating that the species difference was not statistically significant (Table 2).

**TABLE 2:** Comparative seroprevalence and univariable/multivariable odds ratios of *Brucella* antibodies in goats and sheep.

Small ruminant	No. samples	RBPT positive (%) [95% CI]	CFT confirmed (%) [95% CI]	Crude OR (COR) [95% CI]	Fisher’s exact P	Adjusted OR (AOR)^∗^ [95% CI]	Model *p*‐value
Goats (caprine)	77	3 (3.90%) [0.81–10.97]	2 (2.60%) [0.32–9.07]	1.00 (Reference)	—	1.00 (Reference)	—
Sheep (ovine)	63	5 (7.94%) [2.63–17.56]	4 (6.35%) [1.76–15.47]	2.54 [0.45–14.36]	0.41	2.48 [0.42–14.62]	0.33
Total	140	8 (5.71%) [2.50–10.95]	6 (4.29%) [1.59–9.09]	—	—	—	—

^∗^Adjusted for sex and age using Firth’s penalized likelihood multivariable logistic regression.

### 3.3. Seroprevalence of *Brucella* Antibodies Stratified by Demographics

Stratification by sex revealed a higher CFT seroprevalence in females (4.95%, 95% CI: 1.63%–11.18%) than in males (2.56%, 95% CI: 0.06%–13.48%), with a COR of 1.98 (95% CI: 0.22 to 17.50; Fisher’s exact *p* = 0.74) and an AOR of 1.81 (95% CI: 0.20 to 16.35; penalized likelihood *p* = 0.60). By age category, all 6 seropositive cases were restricted to adult animals older than 18 months (6.74%, 95% CI: 2.51%–14.10%), whereas no cases were detected among young animals aged 6–18 months (0.00%, 95% CI: 0.00%–6.98%). Using a 0.5 continuity correction, the unadjusted COR for young versus adult animals was 0.12 (95% CI: 0.01 to 2.26; Fisher’s exact *p* = 0.08). In the multivariable model, the AOR for young animals was 0.14 (95% CI: 0.01 to 1.84; penalized likelihood *p* = 0.15).

Across administrative wards, CFT seroprevalence was 2.70% (95% CI: 0.07%–14.16%) in Chugwi, 2.94% (95% CI: 0.07%–15.33%) in Turu A (COR = 1.09, 95% CI: 0.07–18.15), 5.88% (95% CI: 0.72%–19.68%) in Turu B (COR = 2.25, 95% CI: 0.19–26.00), and 5.71% (95% CI: 0.70%–19.16%) in Vwang (COR = 2.18, 95% CI: 0.19–25.20), with no statistically significant geographical variation (Fisher’s exact *p* = 0.80) (Table 3).

**TABLE 3:** Stratification of *Brucella* antibody seroprevalence across ruminant sex, age, and location.

Demographic variable	Total sample (G vs. S)	RBPT positive (%) [95% CI]	CFT confirmed (%) [95% CI]	Crude OR (COR) [95% CI]	Exact P	Adjusted OR (AOR)^∗^ [95% CI]	Model P
*Sex*							
Female	101 (51 vs. 50)	6 (5.94%) [2.21–12.48]	5 (4.95%) [1.63–11.18]	1.98 [0.22–17.50]	0.74	1.81 [0.20–16.35]	0.6
Male	39 (26 vs. 13)	2 (5.13%) [0.63–17.32]	1 (2.56%) [0.06–13.48]	1.00 (Reference)	—	1.00 (Reference)	—

*Age*							
Young (6–18 months)	51 (25 vs. 26)	1 (1.96%) [0.05–10.45]	0 (0.00%) [0.00–6.98]	0.12 [0.01–2.26]^†^	0.08	0.14 [0.01–1.84]	0.15
Adult (> 18 months)	89 (52 vs. 37)	7 (7.87%) [3.22–15.54]	6 (6.74%) [2.51–14.10]	1.00 (Reference)	—	1.00 (Reference)	—

*Location (ward)*							
Chugwi	37 (20 vs. 17)	2 (5.41%) [0.66–18.19]	1 (2.70%) [0.07–14.16]	1.00 (Reference)	0.80^‡^	—	—
Turu A	34 (19 vs. 15)	1 (2.94%) [0.07–15.33]	1 (2.94%) [0.07–15.33]	1.09 [0.07–18.15]	—	—	—
Turu B	34 (18 vs. 16)	3 (8.82%) [1.86–23.68]	2 (5.88%) [0.72–19.68]	2.25 [0.19–26.00]	—	—	—
Vwang	35 (20 vs. 15)	2 (5.71%) [0.70–19.16]	2 (5.71%) [0.70–19.16]	2.18 [0.19–25.20]	—	—	—

*Note:* Exact P from two‐sided Fisher’s exact test. Model P from Firth’s penalized likelihood logistic regression.

Abbreviation: G versus S = goats versus sheep.

^∗^AOR adjusted simultaneously for species, sex, and age.

^†^Calculated using Haldane–Anscombe 0.5 continuity correction due to zero cell count.

^‡^Overall Fisher’s exact test across the four wards.

## 4. Discussion

This study evaluated the seroprevalence of *Brucella* antibodies among apparently healthy sheep and goats in Vwang District, Central Nigeria. By focusing on clinically healthy animals screened via RBPT and confirmed using CFT, our findings provide specific baseline data regarding *Brucella* antibody presence in nonvaccinated small ruminant flocks. The overall CFT‐confirmed seroprevalence of 4.29% (6/140) demonstrates the presence of circulating *Brucella* antibodies in small ruminants in Vwang District despite the absence of overt clinical signs. When comparing our results with prior literature from Nigeria, our observed seroprevalence (4.29%) is lower than rates reported in broader regional surveys, such as 14.5% in sheep and 16.1% in goats in Plateau State [20], 19.8% in Nasarawa State [21], and 26.5% in Kaduna State [22]. These differences likely stem from methodological and sampling variations; while earlier studies evaluated slaughterhouse populations or herds with active abortion histories, our survey strictly evaluated non‐aborting, clinically healthy animals in a single rural district and applied CFT confirmation to eliminate screening false‐positives.

Although seroprevalence was higher in sheep (6.35%) than in goats (2.60%), this difference was not statistically significant in either univariable analysis or after multivariable adjustment for sex and age. This pattern of higher ovine seropositivity aligns with comparative investigations in similar agro‐pastoral environments in Nepal [23], but contrasts with findings from pastoral regions of Ethiopia where caprine herds predominated [24]. In Vwang District, sheep are frequently grazed on communal pastures alongside cattle (a recognized reservoir for *B. abortus* [25]), whereas goats are predominantly managed in localized household enclosures. While communal grazing represents a plausible transmission pathway [26], grazing contact rates and molecular pathogen lineages (*B. melitensis* vs. *B. abortus*) were not quantitatively evaluated in this survey; hence, this species‐level pattern remains an epidemiological hypothesis requiring validation through future longitudinal studies.

Regarding age, *Brucella* antibodies were detected exclusively in adult animals older than 18 months (6.74%) compared with young animals (0.00%). Because of the zero cell count in young animals, a 0.5 continuity correction was applied in the univariable analysis, and penalized likelihood was employed in the multivariable model. Although not statistically significant, the restriction of seropositivity to sexually mature animals is biologically plausible and consistent with the findings of Bugeza et al. [27] and Natesan et al. [28], who noted that older livestock experience cumulative lifetime exposure risks.

Sex‐stratified analysis showed a higher seroprevalence in females (4.95%) than in males (2.56%), although the association was nonsignificant in both univariable and multivariable analyses. This pattern is consistent with reports by Ukwueze et al. [29] and Dahourou et al. [30], who observed that female small ruminants are typically retained longer within breeding flocks for lactation and reproduction, thereby extending their potential exposure window. Geographical variation across administrative wards was similarly nonsignificant, with seroprevalence ranging from 2.70% in Chugwi to 5.88% in Turu B, likely reflecting localized flock movements and shared watering points.

From a diagnostic perspective, our two‐tier testing strategy highlights the necessity of confirmatory serology. RBPT screening yielded 8 reactive samples (5.71%), but CFT confirmed 6 cases (4.29%), resolving two nonspecific screening reactions. Nonspecific RBPT reactions frequently occur due to serological cross‐reactivity with other Gram‐negative bacteria (such as *Y. enterocolitica* O:9, *Escherichia coli* O157, or *Salmonella* species) that possess shared O‐polysaccharide side‐chain epitopes [31, 32]. Employing CFT as a secondary confirmatory assay substantially enhanced diagnostic specificity [33]. The detection of *Brucella* antibodies in clinically healthy animals provides serological evidence of exposure within smallholder flocks in Vwang District. However, because serological assays detect circulating antibodies rather than viable bacteria [34], these findings indicate prior or current immunological contact rather than confirming active bacterial shedding or bacteremia. These baseline observations underscore the importance of proactive veterinary measures, including routine surveillance, flock biosecurity, and public health education regarding safe milk handling and animal husbandry in rural agrarian communities.

Several methodological and statistical limitations must be acknowledged. First, the study’s cross‐sectional design and reliance on serology mean that antibody detection reflects historical or present immunological exposure to smooth *Brucella* species rather than confirming active bacterial shedding, bacteremia, or culture‐confirmed infection. Second, while individual‐level reproductive history (such as recent abortion) was assessed during presampling screening to exclude clinically affected animals, lifetime reproductive parameters (such as parity or cumulative breeding events) were not recorded as continuous or ordinal variables for risk‐factor modeling. Third, while the sample size of 140 animals provided adequate absolute precision (±3.4%) for baseline prevalence estimation across the district, it had limited statistical power to detect small effect sizes in subgroup comparisons, leading to wide CIs. Future large‐scale, multidistrict surveys that capture comprehensive lifetime reproductive histories alongside bacteriological isolation and molecular (PCR) typing are warranted to definitively identify circulating *Brucella* species (*B. melitensis* vs. *B. abortus*) and quantify transmission dynamics.

## 5. Conclusion

This study provides baseline serological evidence of *Brucella* exposure among apparently healthy sheep (6.35%) and goats (2.60%) in Vwang District, Central Nigeria, using a serial testing protocol (RBPT screening followed by CFT confirmation). Although differences across species, age, sex, and location were not statistically significant in univariable or multivariable analyses, the detection of specific antibodies in nonvaccinated, clinically healthy livestock indicates that *Brucella* exposure occurs within smallholder flocks. These findings support the consideration of routine serological monitoring and community education on safe milk handling and animal hygiene as proactive, preventive veterinary and public health measures in rural agrarian communities.

## Author Contributions

Conceptualization, Martin Zang Pam and Alex Odoom; methodology, Martin Zang Pam, Alex Odoom, Charles Matthew Kakka, Fidelis Leo Latkal, Kete Clinton Waziri, Solomon Jere Dule, Suzanna Zakka Nyango, and Pedung Raphael Emmanuel; software, Martin Zang Pam and Alex Odoom; validation, Martin Zang Pam, Alex Odoom, Charles Matthew Kakka, Fidelis Leo Latkal, Kete Clinton Waziri, Solomon Jere Dule, Suzanna Zakka Nyango, and Pedung Raphael Emmanuel; formal analysis, Martin Zang Pam and Alex Odoom; investigation, Martin Zang Pam, Alex Odoom, Charles Matthew Kakka, Fidelis Leo Latkal, Kete Clinton Waziri, Solomon Jere Dule, Suzanna Zakka Nyango, and Pedung Raphael Emmanuel; resources, Martin Zang Pam, Alex Odoom, Charles Matthew Kakka, Fidelis Leo Latkal, Kete Clinton Waziri, Solomon Jere Dule, Suzanna Zakka Nyango, and Pedung Raphael Emmanuel; data curation, Martin Zang Pam, Alex Odoom, Charles Matthew Kakka, Fidelis Leo Latkal, Kete Clinton Waziri, Solomon Jere Dule, Suzanna Zakka Nyango, and Pedung Raphael Emmanuel; writing–original draft preparation, Martin Zang Pam, Alex Odoom, Charles Matthew Kakka, Fidelis Leo Latkal, Kete Clinton Waziri, Solomon Jere Dule, Suzanna Zakka Nyango, and Pedung Raphael Emmanuel; writing–review and editing, Martin Zang Pam, Alex Odoom, Charles Matthew Kakka, Fidelis Leo Latkal, Kete Clinton Waziri, Solomon Jere Dule, Suzanna Zakka Nyango, and Pedung Raphael Emmanuel; visualization, Martin Zang Pam and Alex Odoom; supervision, Martin Zang Pam and Alex Odoom; project administration, Martin Zang Pam Charles Matthew Kakka and Alex Odoom; funding acquisition, Martin Zang Pam and Alex Odoom.

## Funding

No funding was received for this research.

## Disclosure

All authors have read and approved the final version of the manuscript. Alex Odoom had full access to all of the data in this study and takes complete responsibility for the integrity of the data and the accuracy of the data analysis.

The corresponding author, Alex Odoom, affirms that this manuscript is an honest, accurate, and transparent account of the study being reported; that no important aspects of the study have been omitted; and that any discrepancies from the study as planned have been explained.

## Ethics Statement

The study’s ethical clearance approval (NVRI/AEC Ref. number: AEC/02/159/24) came from the National Veterinary Research Institute Animal Ethics Committee after the Federal College of Animal Health and Production Technology, Vom, granted permission for conducting the work. All procedures adhered to the International Council for Laboratory Animal Science (ICLAS) [19].

## Consent

The authors have nothing to report.

## Conflicts of Interest

The authors declare no conflicts of interest.

## Data Availability

Data are available on request from the corresponding author.

## References

[1] Qureshi K. A. , Parvez A. , Fahmy N. A. et al., Brucellosis: Epidemiology, Pathogenesis, Diagnosis and Treatment–A Comprehensive Review, Annals of Medicine. (2024) 55, no. 2, 10.1080/07853890.2023.2295398. PMC10769134 38165919

[2] Ghssein G. , Ezzeddine Z. , Tokajian S. et al., Brucellosis: Bacteriology, Pathogenesis, Epidemiology and Role of the Metallophores in Virulence: A Review, Frontiers in Cellular and Infection Microbiology. (2025) 15, 10.3389/FCIMB.2025.1621230/FULL. PMC12279742 40697821

[3] Huy T. X. N. , Nguyen T. T. , Kim H. , Reyes A. W. B. , and Kim S. , Brucella Phagocytosis Mediated by Pathogen-Host Interactions and Their Intracellular Survival, Microorganisms. (2022) 10, no. 10, 10.3390/MICROORGANISMS10102003. PMC9610446 36296279

[4] Głowacka P. , Zakowska D. , Naylor K. , Niemcewicz M. , and Bielawska-Drózd A. , Brucella–Virulence Factors, Pathogenesis and Treatment, Polish Journal of Microbiology. (2018) 67, no. 2, 151–161, 10.21307/PJM-2018-029. PMC7256693 30015453

[5] Tulu D. , Bovine Brucellosis: Epidemiology, Public Health Implications, and Status of Brucellosis in Ethiopia, Veterinary Medicine: Research and Reports. (2022) 13, 21–30, 10.2147/VMRR.S347337. PMC8752066 35028300

[6] Khurana S. K. , Sehrawat A. , Tiwari R. et al., Bovine Brucellosis–A Comprehensive Review, Veterinary Quarterly. (2021) 41, no. 1, 61–88, 10.1080/01652176.2020.1868616. PMC7833053 33353489

[7] Brunetti R. , Ottaiano M. , Fordellone M. et al., Risk Factors for the Spread of Brucellosis in Sheep and Goats in the Campania Region in the Years 2015–2020, Microorganisms. (2023) 11, 10.3390/MICROORGANISMS11112623. PMC10673264 38004635

[8] Saxena N. , Singh B. B. , and Saxena H. M. , Brucellosis in Sheep and Goats and Its Serodiagnosis and Epidemiology, International Journal of Current Microbiology and Applied Sciences. (2018) 7, no. 1, 1848–1877, 10.20546/IJCMAS.2018.701.225.

[9] Rossetti C. A. , Maurizio E. , and Rossi U. A. , Comparative Review of Brucellosis in Small Domestic Ruminants, Frontiers in Veterinary Science. (2022) 9, 10.3389/FVETS.2022.887671. PMC9133814 35647101

[10] Di Febo T. , Di Francesco G. , Grattarola C. et al., Serological Diagnosis of Brucella Infection in Cetaceans by Rapid Serum Agglutination Test and Competitive ELISA With Brucella Abortus and Brucella Ceti as Antigens, Pathogens. (2025) 14, no. 1, 10.3390/PATHOGENS14010026/S1. PMC11768795 39860987

[11] Songül Ö. , Ahmet M. , and Ayfer G. , Evaluation of the Use of Lipopolysaccharides and Synthetic O-Polysaccharides in I-ELISA for the Serological Diagnosis of Brucellosis, German Journal of Microbiology. (2025) 5, no. 2, 27–32, 10.51585/GJM.2025.2.0051.

[12] Ötkün S. , Gürbilek S. E. , Yücetepe A. G. , Tel O. Y. , and Keskin O. , Serological Evaluation of Brucellosis in Nomadic Small Ruminants in Southeastern Türkiye, BioMed Central Veterinary Research. (2026) 22, 10.1186/S12917-026-05372-4. PMC13088734 41814345

[13] Vection S. , Laine C. G. , and Arenas-Gamboa A. M. , What do we Really Know About Brucellosis Diagnosis in Livestock Worldwide? a Systematic Review, Public Library of Science Neglected Tropical Diseases. (2025) 19, no. 6, 10.1371/JOURNAL.PNTD.0013185. PMC12173231 40526752

[14] Aworh M. K. , Okolocha E. C. , Awosanya E. J. , and Fasina F. O. , Sero-Prevalence and Intrinsic Factors Associated With Brucella Infection in Food Animals Slaughtered at Abattoirs in Abuja, Nigeria, BioMed Central Research Notes. (2017) 10, no. 1, 10.1186/S13104-017-2827-Y. PMC5634886 29017544

[15] Maurice N. A. , Wungak S. Y. , Gana B. A. et al., Seroprevalence of Bovine Brucellosis in Northern Plateau State, North Central Nigeria, Asian Pacific Journal of Tropical Disease. (2013) 3, no. 5, 337–340, 10.1016/S2222-1808(13)60081-X.

[16] Legesse A. , Mekuriaw A. , Gelaye E. et al., Comparative Evaluation of RBPT, I-ELISA, and CFT for the Diagnosis of Brucellosis and PCR Detection of Brucella Species From Ethiopian Sheep, Goats, and Cattle Sera, BioMed Central Microbiology. (2023) 23, no. 1, 10.1186/S12866-023-02962-2. PMC10413706 37563597

[17] Alton G. G. , Jones L. M. , Pietz D. E. , and Organization W. H. , Nations F and AO of the U. Laboratory Techniques in Brucellosis, 1975, https://iris.who.int/handle/10665/38676. 812265

[18] WOAH , Reference Reagents-WOAH-World Organisation for Animal Health, https://www.woah.org/en/what-we-offer/quality-veterinary-products/reference-reagents/.

[19] ICLAS , ICLAS [Internet], https://iclas.org/.

[20] Bertu W. J. , Ajogi I. , Bale J. O. O. , Kwaga J. K. P. , and Ocholi R. A. , Sero-Epidemiology of Brucellosis in Small Ruminants in Plateau State, Nigeria, African Journal of Microbiology Research. (2010) 4, no. 19, 1935–1938, 10.5897/AJMR.9000650.

[21] Agada C. A. , Ogugua A. J. , and Anzaku E. J. , Occurrence of Brucellosis in Small Ruminants Slaughtered in Lafia Central Abattoir, Nasarawa State, Nigeria, Sokoto Journal of Veterinary Sciences. (2018) 16, no. 1, 10.4314/SOKJVS.V16I1.3.

[22] Kaltungo B. Y. , Saidu S. N. A. , Sackey A. K. B. , and Kazeem H. M. , Sero-Prevalence of Brucellosis in Sheep in North Senatorial District of Kaduna State, Nigeria, Asian Pacific Journal of Tropical Disease. (2015) 5, no. 2, 163–168, 10.1016/S2222-1808(14)60646-0.

[23] Gompo T. R. , Shah R. , Tiwari I. , and Gurung Y. B. , Sero-Epidemiology and Associated Risk Factors of Brucellosis Among Sheep and Goat Population in the South Western Nepal: A Comparative Study, BioMed Central Veterinary Research. (2021) 17, 10.1186/S12917-021-02835-8. PMC7992955 33766040

[24] Getachew A. , De Massis F. , Mekonnen B. et al., Seroprevalence, Characterization, and Risk Factors of Brucellosis in Cattle, Sheep, Goats, and Camels in the Oromia Region, Borena Zone, Ethiopia, Preventive Veterinary Medicine. (2025) 244, 10.1016/J.PREVETMED.2025.106634. 40729977

[25] Lita E. P. , Mkupasi E. M. , Ochi E. B. et al., Molecular Evidence of Brucella Abortus Circulating in Cattle, Goats, and Humans in Central Equatoria State, South Sudan, Scientific Reports. (2025) 15, no. 1, 10.1038/s41598-025-87368-y. PMC11986056 40210670

[26] Teshome D. , Sori T. , Banti T. , Kidane G. , Wieland B. , and Alemayehu G. , Prevalence and Risk Factors of Brucella spp. in Goats in Borana Pastoral Area, Southern Oromia, Ethiopia, Small Ruminant Research. (2022) 206, 10.1016/J.SMALLRUMRES.2021.106594.

[27] Bugeza J. , Roesel K. , Moriyon I. et al., Sero-Prevalence and Factors Associated With Anti-Brucella Antibodies in Slaughter Livestock in Uganda, Frontiers in Epidemiology. (2023) 3, 10.3389/FEPID.2023.1213592/TEXT. PMC10910896 38455915

[28] Natesan K. , Kalleshamurthy T. , Nookala M. et al., Seroprevalence and Risk Factors for Brucellosis in Small Ruminant Flocks in Karnataka in the Southern Province of India, Veterinary World. (2021) 14, no. 11, 2855–2862, 10.14202/VETWORLD.2021.2855-2862. PMC8743770 35017831

[29] Ukwueze K. O. , Ishola O. O. , Dairo M. D. , Awosanya E. J. , and Cadmus S. I. , Seroprevalence of Brucellosis and Associated Factors Among Livestock Slaughtered in Oko-Oba Abattoir, Lagos State, Southwestern Nigeria, Pan African Medical Journal. (2020) 36, 1–11, 10.11604/PAMJ.2020.36.53.21094. PMC7392031 32774628

[30] Dahourou L. D. , Ouoba L. B. , Minoungou L. B. G. et al., Prevalence and Factors Associated With Brucellosis and Tuberculosis in Cattle From Extensive Husbandry Systems in Sahel and Hauts-Bassins Regions, Burkina Faso, Scientific African. (2023) 19, 10.1016/J.SCIAF.2023.E01570.

[31] Bonfini B. , Chiarenza G. , Paci V. et al., Cross-Reactivity in Serological Tests for Brucellosis: A Comparison of Immune Response of Escherichia Coli o157:H7 and Yersinia Enterocolitica o:9 Vs Brucella Spp, Veterinaria Italiana. (2018) 54, no. 2, 107–114, 10.12834/VETIT.1176.6539.2. 30019327

[32] Bányász B. , Antal J. , and Dénes B. , False Positives in Brucellosis Serology: Wrong Bait and Wrong Pond?, Tropical Medicine and Infectious Disease. (2023) 8, no. 5, 10.3390/TROPICALMED8050274. PMC10220701 37235322

[33] Vicente A. F. , Troupin C. , Grayo S. et al., Comparison of Serological Tools for Reliable Diagnosis of Brucellosis Circulation in the West-African Context, BMC Veterinary Research. (2026) 22, no. 1, 10.1186/S12917-025-05183-Z. PMC12911309 41508007

[34] Abnaroodheleh F. , Ansari F. , Shahali Y. , and Dadar M. , Diagnostic Performance of Four Serological Assays for Bovine Brucellosis and Optimised Cutoff Thresholds in an Endemic Region of Iran, Veterinary Medicine and Science. (2025) 11, no. 5, 10.1002/VMS3.70566. PMC12352339 40810840

